# The role of microenvironment in the initiation and evolution of B-cell precursor acute lymphoblastic leukemia

**DOI:** 10.3389/fonc.2023.1150612

**Published:** 2023-03-07

**Authors:** Alicia Garcia-Gimenez, Simon E. Richardson

**Affiliations:** ^1^ Department of Haematology, Wellcome Trust—Medical Research Council Cambridge Stem Cell Institute, University of Cambridge, Cambridge, United Kingdom; ^2^ Cambridge University Hospitals, Cambridge, United Kingdom

**Keywords:** microenvironment, tumor evolution, chemoresistance, epigenetics, B- cell precursor acute lymphoblastic leukemia (BCP-ALL)

## Abstract

B cell precursor acute lymphoblastic leukemia (BCP-ALL) is a malignant disorder of immature B lineage immune progenitors and is the commonest cancer in children. Despite treatment advances it remains a leading cause of death in childhood and response rates in adults remain poor. A preleukemic state predisposing children to BCP-ALL frequently arises *in utero*, with an incidence far higher than that of transformed leukemia, offering the potential for early intervention to prevent disease. Understanding the natural history of this disease requires an appreciation of how cell-extrinsic pressures, including microenvironment, immune surveillance and chemotherapy direct cell-intrinsic genetic and epigenetic evolution. In this review, we outline how microenvironmental factors interact with BCP-ALL at different stages of tumorigenesis and highlight emerging therapeutic avenues.

## Introduction

Cancer is a clonal outgrowth of cells that have adapted to gain a competitive advantage over their physiologically-constrained competitors. Classically this has been viewed as a stepwise evolutionary process, characterized by the sequential acquisition of driver mutations in critical tumor suppressor and oncogenes. However, powerful non-genetic factors also direct and constrain tumor evolution, including cell-intrinsic “epigenetic” states acquired from the developmental origins, stem/progenitor cell programming and epigenetic dysregulation of the cell of origin, and cell-extrinsic factors, notably microenvironmental niches and the selective pressure of immune surveillance and therapy. In this review we will outline how cell-extrinsic factors contribute to the initiation and evolution of B-cell precursor acute lymphoblastic leukemia (BCP-ALL), highlighting opportunities for therapeutic intervention at different stages of disease progression and chemoresistance.

## B-Cell precursor acute lymphoblastic leukemia

BCP-ALL is an aggressive malignancy of immature B-lineage immune cell progenitors. Leukemic cells arise in the bone marrow (BM) and infiltrate extramedullary sites, notably the reticuloendothelial system (liver, spleen & lymph nodes) and so-called sanctuary sites (central nervous system (CNS) and testes) (1). BCP-ALL is predominantly a disease of childhood and there is compelling evidence in a number of genetic subgroups that a pre-leukemic state is initiated *in utero*, with B-cells harboring clonal genetic fusions or immunoglobulin gene rearrangements identifiable at birth on neonatal blood samples from affected children and/or shared in the blood of monochorionic twins (2). Importantly, the incidence of pre-leukemia in children far exceeds that of overt disease, indicating that these cells require further genetic and/or other events to transform to frank leukemia.

The first hit mutations that can initiate pre-leukemia are highly diverse between patients, but recent work has shown that the disease can be classified into 23 distinct subgroups based on underlying transcriptional signatures (3, 4). In contrast, the second-hit mutations implicated in disease transformation and progression are relatively conserved across genetic subtypes, implicating common pathways in disease progression including activation of signaling pathways (e.g. *RAS*, *JAK-STAT*), loss of transcriptional master regulators (e.g. *PAX5, IKZF1*) and perturbation of epigenetic co-regulators (e.g. *CREBBP*) (3–6).

Modern response-adapted multiagent chemotherapy regimens can cure the majority of children, albeit at the expense of toxicity (7, 8). Treatment of adult B-ALL, however, is more challenging with approximately 50% overall survival even in patients fit enough to undergo allogeneic bone marrow transplantation (9, 10). Introduction of novel targeted therapies (e.g. BCR::ABL1 tyrosine kinase inhibitors) and immunotherapy (e.g. CD19-CD3 bispecific T cell engager (BiTEs) and chimeric antigen receptor (CAR)-T cells) are improving outcomes for certain high-risk and relapsed cases. Nevertheless, some groups of patients continue to fare poorly, including those with high-risk genetic drivers (e.g. *E2A::HLF*, *KMT2A*-rearranged BCP-ALL), certain age groups (e.g. infants, elderly), those with poor-risk second hit mutations (e.g. *CREBBP-RAS*) and those who relapse in the CNS.

**Figure 1:**
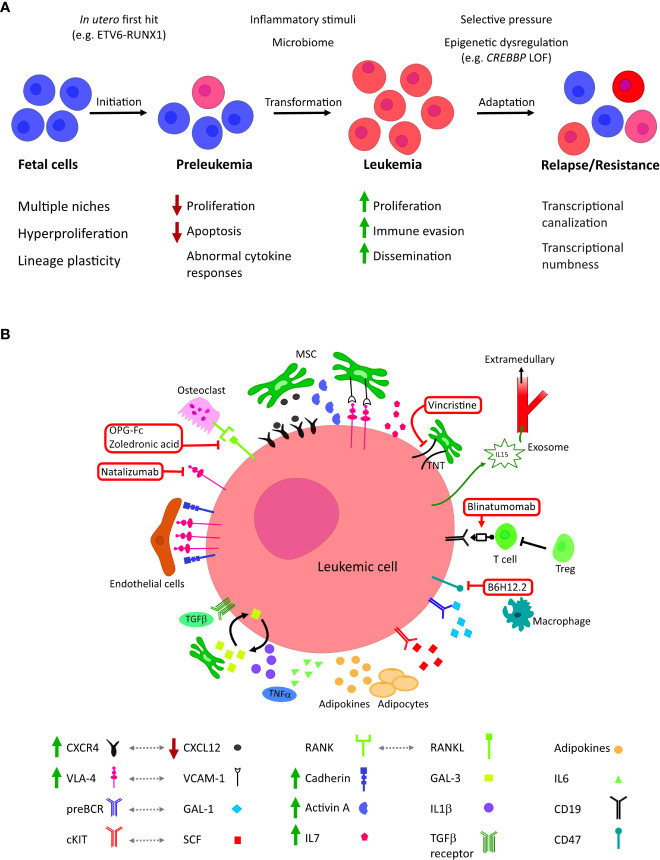
**(A)** The roles of microenvironmental factors at different stages of tumor evolution in childhood BCP-ALL. BCP-ALL is thought to initiate *in utero* where B lineage progenitors exhibit unique characteristics, including inhabiting diverse niches. First-hit mutations create a clinically silent preleukemic state that persists into childhood. In a small number of children, this pre leukemic state transforms to acute leukemia; this is thought to be driven by the acquisition of co-operative second hit mutations, possibly in response to inflammatory signaling. Leukemic blasts evolve to colonize further niches and evade treatment, altering their epigenetic state to become less dependent on external stimuli. **(B)** Cellular and molecular microenvironmental factors associated with BCP-ALL. Current and investigational therapeutic targets are highlighted in red: mitochondrial transfer through tunnelling nanotubules (TNT) can be inhibited by the microtubule inhibitor vincristine; blinatumomab co-associates B-lineage blasts with tumour-targeting cytotoxic T cells; neutralization of CD47 by B6H12.2 antibody restores the macrophage phagocytic response against leukemic blasts; zoledronic acid and recombinant OPG-Fc reduce bone loss, inhibiting leukemic growth and prolonging survival *in vivo*; integrin 4 inhibitor Natalizumab sensitizes leukemic blasts to chemotherapy.

Microenvironmental and cell-extrinsic factors are increasingly thought to play a both supportive and constraining roles throughout the pathogenesis of BCP-ALL (**Figure 1A**). Understanding how they contribute to pre-leukemic initiation, the fate of preleukemic clones and mechanisms of resistance will offer new avenues for understanding disease mechanism and facilitate the development of novel therapeutic interventions.

## Niche in normal B cell development

The BM niche is formed by hematopoietic and non-hematopoietic cells from different lineages. Bone forming cells (osteoblasts and osteoclasts), adipocytes, reticular cells, endothelial cells, mesenchymal stromal cells (MSC) and neural cells help create and support BM homeostasis and hematopoiesis, through direct cellular interactions, the production of soluble cytokines and the maintenance of the extracellular matrix (11–13). Interactions between hematopoietic stem/progenitor cells (HSPCs) and the BM microenvironment are essential for both commitment into the B lineage and survival following successful rearrangement of the heavy and light chain immunoglobulin loci, evaluated by pro-survival signaling through the pre-B cell receptor (preBCR) and BCR complexes respectively. A number of soluble factors are known to be essential for the commitment into the B lineage, including FLT3L and SCF (14, 15). Multipotent hematopoietic progenitor cells and early pre-proB cells directly co-associate with CXCL12-expressing stromal cells (16), with osteoblasts appearing particularly important for successful B lineage differentiation (17). CXCL12 seems important in both attracting B progenitors to specific BM niches and by inducing direct interactions through the integrin VLA-4 - VCAM1 axis (18).

Different parts of the BM niche exhibit different metabolic characteristics, in particular oxygenation, which is thought to regulate REDOX-sensitive transcription factors such as the hypoxia inducible factors as well as the B cell master regulator PAX5 (19). Maturing proB cells migrate to areas of high IL7 expression and complete RAG-mediated rearrangement of their *IGH* locus to become preB cells, which express the preBCR. Successful recombination of the *IGH* locus results in effective signaling through the preBCR, which is in part activated by stromal Galectin 1 binding (20). Successful light chain recombination completes BM B cell maturation, producing a repertoire of naïve B cells with a unique BCR competent for antigenic stimulation in lymph nodes.

B cell progenitors are particularly abundant during early embryonic development. During ontogeny hematopoiesis arises in multiple waves from diverse sites, including primitive hematopoiesis in the yolk sac (YS) before definitive HSCs are specified in the aorto-gonad-mesonephros (AGM) region of the dorsal aorta. The progeny of these HSPCs establish differentiation hierarchies in multiple niches, notably the YS, fetal liver (FL), placenta and BM (21). The precise lineages of the B cell progenitors produced in these sites remains controversial (22), but it appears that both the ontogenic origins and the niche in which these cells reside are associated with significant differences to adult lymphopoiesis, in particular the enhanced proliferative state of FL HSPCs (23, 24). These differences indicate that fetal hematopoiesis may be structured differently to adult, providing a unique cellular context for the *in utero* initiation of childhood leukemia.

## Subversion of B-cell-microenvironmental interactions promotes pre-leukemic development

There is evidence that pre-leukemic cells are supported by the microenvironment following disease initiation and that the microenvironment becomes remodeled to permit and support disease progression (25) (**Figure 1B**).

Much work has been undertaken in the paradigmatic pre-leukemia initiated *in utero* by the *ETV6::RUNX1* gene fusion, the commonest single genetic cause of childhood BCP-ALL. Pre-leukemic *in vitro* model systems expressing the first-hit mutation show aberrant co-expression of myeloid and erythroid cytokine receptors on lymphoid cells, indicating that even at the earliest stages, pre-leukemic cells significantly change the way that they sense and respond to their microenvironment (26–28). Functionally, preleukemic cells are thought to be maintained as a small population, with niche factors contributing to their maintenance in a relatively quiescent (26, 29) and anti-apoptotic state (30).

Prenatally, the major site of pre-leukemia is likely to be the FL, although direct transplacental exposure to mutagens has been implicated in the generation of first-hit mutations, and by the time of birth pre-leukemic cells are readily detectable in the peripheral blood and umbilical cord. HSCs emerging from the AGM are attracted to and retained in the FL niche by factors including SCF, CXCL12 and β1 integrin, factors which are also supportive of B cell development (31, 32). Compared to both adult and fetal BM, FL hematopoiesis is characterized by extremely high levels of proliferation (33), along with other differences in cell surface markers, lineage differentiation and gene expression profiles (23, 34). B-lymphopoiesis is particularly prominent in the FL and exhibits a number of qualitative differences to adult including lack of *TDT* expression, IL7R independence and that the earliest committed B cells appear to emerge from a developmentally-restricted progenitor that exhibits unique co-expression of myeloid and lymphoid programming and potential (28). These differences might explain some of the unique features of childhood BCP-ALL, including its relatively high incidence, propensity for unique genetic drivers rarely seen in adults (e.g. *ETV6::RUNX1*, hyperdiploidy) and tendency to lineage promiscuity.

By the end of gestation, it is probable that pre-malignant cells have colonized the BM. There is evidence that multiple first-hit mutations can change B cell adhesion/migration properties; for example, ETV6::RUNX1 is associated with a cell-intrinsic defect in CXCR4-CXCL12 signaling (35) and *KMT2A*-mutated BCP-ALL up-regulates protocadherin genes (36). An area of active interest is whether pre-leukemic cells can modify the BM niche prior to transformation; given the changes seen at the time of diagnosis (see below) this seems likely and novel experimental co-culture techniques will help in delineating these interactions.

## Inflammatory stimuli aid the transformation and maintenance of overt leukemia

The incidence of detectable pre-leukemia in asymptomatic children is many times that of the incidence of overt childhood BCP-ALL; for example, *ETV6::RUNX1^+^* pre-leukemia is approximately 100-500x more common than *ETV6::RUNX1^+^* BCP-ALL (37, 38). This indicates the importance of second-hit events in driving disease transformation, thought mainly to be the acquisition of co-operative mutations. Conversely, it demonstrates that the majority of pre-leukemic clones in children lack sufficient self-renewal potential to persist into adulthood, either in a cell-intrinsic manner, or due to an inability to adapt to changing niches during development (39).

Epidemiological evidence points to delayed exposure to infectious stimuli as a risk factor for BCP-ALL transformation. This has led to the hypothesis that an under-exposed immune system in early life results in subsequent overactivation of cytokine signaling and hyper-mutagenesis in pre-malignant cells, potentially driven by aberrant activation-induced cytidine deaminase (AID) (40) or recombination-activating gene (RAG) activity (41, 42). Furthermore, the neonatal blood of children who develop leukemia exhibits measurable differences in cytokine concentrations, indicating a degree of immune dysregulation prior to environmental exposures to pathogens (43, 44). The specific pathogens involved in this mechanism are unknown, but significant epidemiological associations with outbreaks of swine flu (45), influenza (46) and SARS (47) viruses have been reported. The effects of both exposure to Covid19 and the lack of infectious exposure in children during prolonged lockdowns are an area of active research (48).

Experimental evidence shows that pre-leukemic mouse models housed in clean specified pathogen free facilities develop increased rates of leukemia in response to infectious stimuli (49, 50) and that inhibition of inflammatory stimulation can mitigate transformation to BCP-ALL in a *Pax5* pre-leukemic model (51). There is evidence that MSC-derived pro-inflammatory TGFβ family members (including TGFβ and Activin A) can favor the growth of *ETV6::RUNX1^+^* pre-leukemic cells (26, 52) and that the acute-phase response cytokines (IL6, TNFα and IL1β) can co-operate with MSCs to generate a pro-leukemic niche for *ETV6::RUNX1^+^* cells and drive a hyper-mutagenic state (53). Intriguingly, gut commensal microbes are known to affect the immune microenvironment systemically and it has been shown in murine pre-leukemic models that microbiome disruption by antibiotic administration in early life can induce the development of leukemia (54).

Once transformed, it is established that BCP-ALL actively remodels its interaction with the BM niche into a permissive or supportive microenvironment (55). The BCP-ALL-MSC interaction appears key to this, with down-regulation of the CXCL12 chemokine-axis favoring BCP-ALL MSC interactions over those of normal HSCs (56). Subversion of a number soluble and surface ligand axes has been implicated in promoting leukemic survival, including TGFβ, Cadherin-wnt-Catenin, Notch and Integrin pathways. Furthermore, BCP-ALL cells inhibit osteoblast function through the RANK-RANKL axis, remodeling the BM space and contributing to clinically-meaningful trabecular bone destruction (57). Zoledronic acid (58) and the recombinant RANKL antagonist, OPG-Fc (59) have shown efficacy in restoring bone homeostasis and reducing disease burden in *in vivo* models. MSC-derived Galectin 3 can be internalized by BCP-ALL blasts stimulating autocrine production of Galectin 3, driving disease progression in a cell autonomous manner (60). This serves as an example of how leukemic cells can also adapt to circumvent their own dependency on microenvironmental factors.

## Epigenetic adaptation to extracellular cues underlies tumor evolution and resistance

During tumor evolution BCP-ALL subclones increasingly compete with each other, adapting to diversify their signaling, metabolic and niche dependencies, as well as evading external pressures including immune surveillance and chemotherapy.

In experimental models, interactions between BCP-ALL and factors from BM perivascular, endosteal, and hematopoietic niches maintain BCP-ALL survival and quiescence in response to chemotherapy (61). A preclinical *in vivo* model has shown that targeting the integrin VLA-4 using the therapeutic antibody Natalizumab sensitized BCP-ALL cells to cytotoxic chemotherapy (62). A fascinating mechanism of chemoresistance has been demonstrated by Burt and colleagues, who showed that direct connections between BCP-ALL blasts and MSCs by tunneling nanotubules (TNT) could lead to mitochondrial transfer, protecting BCP-ALL cells from toxicity from chemotherapy-induced reactive oxygen species (63). The microtubule inhibitor vincristine, a key component of current BCP-ALL chemotherapy regimens, was shown to disrupt TNT formation, ameliorating this mechanism of resistance. Adipocytes have been shown to induce chemoresistance by both directly sequestering chemotherapeutic agents and by increasing leukemic blasts fitness through the production of pro- and anti-inflammatory adipokines, fueling the leukemic cells with free fatty acids and promoting oxidative phosphorylation (64, 65). The upregulation of Galectin-9 by adipokines is responsible for several of these effects with preliminary studies implicating Galectin-9 as a therapeutic target (66). Interestingly, the role of adipocytes has been shown to alter dynamically during treatment, with a particular role in promoting a quiescent chemo-resistant state in regenerating bone marrow. Mechanistically this was associated with a global suppression of protein translation, which could be overcome by inhibition of GCN2, restoring chemosensitivity (67).

The established graft-versus-leukemia effects seen after allogeneic transplantation, and more recently the advent of highly-efficacious BiTE and CAR-T immunotherapies in BCP-ALL, highlights the necessity for continuous T cell immune evasion during leukemic evolution. The efficacy of the CD19-CD3 BiTE blinatumomab demonstrates the ongoing presence of anti-BCP-ALL cytotoxic T cells in frank disease. Blinatumomab failure has been associated with higher numbers of inhibitory Treg cells, implicating these negative regulators of the cellular immune response as potential effectors of immune evasion (68). Furthermore, BCP-ALL blasts overexpress the surface CD47, a “don’t eat me” signal that inhibits phagocytosis by macrophages. The anti-CD47 neutralizing antibody B6H12.2 has been shown to relieve this block in *in vitro* and *in vivo* models and this strategy has shown promise in other leukemia subtypes (69).

Clinically important sites of relapse include the CNS and testes. These are considered sanctuary sites, with demonstrably lower levels of exposure to chemotherapy drugs in addition to being relatively privileged sites from immune surveillance (70–72). Extramedullary niches share an impaired CXCR4-CXCL12 axis (73, 74), hypoxic conditions (75) and the presence of the cytokine SCF (76). B lymphocytes have a physiological ability to enter the CNS and the ability to cross the blood-cerebrospinal fluid barrier is a generic feature of BCP-ALL blasts (77). A number of surface receptors are thought to contribute to this, including classical B-cell progenitor markers such as CD79a and IL7R (78, 79). Leukemic blasts also produce exosomes with soluble molecules (e.g. IL15) that alter distant niches such as the CNS (80). Once in the CNS, leukemic cells adapt to their new environment, including by adjusting to relative hypoxia (75) and by changing their metabolic requirements towards fatty acid metabolism (81, 82), changes that potentially provide unique therapeutic vulnerabilities.

As BCP-ALL progresses, cells appear to become more autonomous, characterized by reduced dependence on external/niche stimuli and the ability to tolerate more extreme environmental and therapeutic selective pressure. This was initially thought to be due to the positive selection of genetically heterogenous subclones, favoring those harboring mutations that provided selective advantages to particular evolutionary pressures. Clinical and experimental studies, however, have failed to demonstrate recurrent selection of genetic sub-clones during treatment, notwithstanding mutations in a small number of direct drug targets (e.g. BCR::ABL1 tyrosine kinase mutations and mutations in key members of the glucocorticoid or mercaptopurine pathways). Instead, leukemic cells appear to adapt to treatment-related selective pressure by “transcriptional canalization”, characterized by increased quiescence and a reduction in their global transcriptional heterogeneity (83). In a genetically highly diverse disease, this acquired loss of heterogeneity at the gene expression level could provide common therapeutic vulnerabilities.

Epigenetic dysregulation is a hallmark of many cancers and the genes encoding epigenetic co-regulators are commonly mutated in BCP-ALL. An intriguing study has recently shown that perturbation of multiple different epigenetic regulators across diverse cancer models tended to increase the tolerance of cancer cells to environmental stress (84). This “transcriptional numbness” to selective pressure lowers the probability of cell death, providing a phenotypic inertia that facilitates survival and adaptation of mutant cells. In the case of BCP-ALL, a potential exemplar of this are loss of function mutations in the transcriptional co-activator *CREBBP*. *CREBBP* deletion, or point mutations affecting its enzymatic acetyltransferase domain, are enriched in relapsed BCP-ALL (6) and high-risk genetic subtypes such as near-haploid BCP-ALL (85). It has also been associated with relapse risk in the otherwise good-risk subgroup of pediatric hyperdiploid BCP-ALL (4, 86). A number of mechanisms could account for this risk, including induction of glucocorticoid resistance (6, 87) and/or potentiation of cytokine signaling though the RAS pathway (88), as well as potentially phenotypic inertia related to loss of transcriptional co-activation. Understanding how cell-intrinsic epigenetic reprograming mediates BCP-ALL adaptation is therefore essential to better appreciating and targeting the cell-extrinsic dependencies seen at different stages of disease progression.

## Discussion

BCP-ALL cells are highly dependent on microenvironmental niches at all stages of tumor development. Characterizing these shifting dependencies is an essential component of undertesting the selective pressure that drives tumor evolution through pre-leukemic initiation, leukemic transformation, frank leukemia, tissue infiltration and chemoresistance. New model systems are emerging to examine these factors, including humanized *in vivo* models and advanced 2D and 3D co-culture systems (89, 90). Perturbation of critical soluble factors, cell-cell and immune interactions are providing promising novel therapeutic avenues, as well as new insights into the mechanism of action of established drugs. Delineating the role that epigenetic reprogramming plays during tumor evolution and treatment resistance will provide opportunities to target the most resistant cases. Conversely, the clear dependencies of early pre-leukemic cells on microenvironmental factors might afford the potential for early intervention and the tantalizing possibility of making childhood BCP-ALL a preventable disease (91).

## Author contributions

AG-G and SR drafted the manuscript. A-GG and SR reviewed and approved the final submission. All authors contributed to the article and approved the submitted version.

## References

[1] Hunger SP Mullighan CG. Acute lymphoblastic leukemia in children. N Engl J Med (2015) 373(16):1541–52. doi: 10.1056/NEJMra1400972 26465987

[2] Ford AM Colman S Greaves M. Covert pre-leukaemic clones in healthy co-twins of patients with childhood acute lymphoblastic leukaemia. Leuk (2022) 37(1):47–52, 1-6. doi: 10.1038/s41375-022-01756-1 PMC9883163 36536099

[3] Gu Z Churchman ML Roberts KG Moore I Zhou X Nakitandwe J. PAX5-driven subtypes of b-progenitor acute lymphoblastic leukemia. Nat Genet (2019) 51(2):296–307. doi: 10.1038/s41588-018-0315-5 30643249 PMC6525306

[4] Brady SW Roberts KG Gu Z Shi L Pounds S Pei D. The genomic landscape of pediatric acute lymphoblastic leukemia. Nat Genet (2022) 54(9):1376–89. doi: 10.1038/s41588-022-01159-z PMC9700506 36050548

[5] Malinowska-Ozdowy K Frech C Schönegger A Eckert C Cazzaniga G Stanulla M. KRAS and CREBBP mutations: A relapse-linked malicious liaison in childhood high hyperdiploid acute lymphoblastic leukemia. Leukemia. (2015) 29:1656–67. doi: 10.1038/leu.2015.107 PMC4530204 25917266

[6] Mullighan CG Zhang J Kasper LH Lerach S Payne-Turner D Phillips LA. CREBBP mutations in relapsed acute lymphoblastic leukaemia. Nat (2011) 471(7337):235–9. doi: 10.1038/nature09727 PMC3076610 21390130

[7] Demidowicz E Pogorzała M Łęcka M Żołnowska H Marjańska A Kubicka M. Outcome of pediatric acute lymphoblastic leukemia: Sixty years of progress. Anticancer Res (2019) 39(9):5203–7. doi: 10.21873/anticanres.13717 31519634

[8] Hunger SP Lu X Devidas M Camitta BM Gaynon PS Winick NJ. Improved survival for children and adolescents with acute lymphoblastic leukemia between 1990 and 2005: A report from the children’s oncology group. J Clin Oncol (2012) 30(14):1663–9. doi: 10.1200/JCO.2011.37.8018 PMC3383113 22412151

[9] Ssenyonga N Stiller C Nakata K Shalkow J Redmond S Bulliard JL. Worldwide trends in population-based survival for children, adolescents, and young adults diagnosed with leukaemia, by subtype, during 2000–14 (CONCORD-3): analysis of individual data from 258 cancer registries in 61 countries. Lancet Child Adolesc Heal (2022) 6(6):409–31. doi: 10.1016/S2352-4642(22)00095-5 35468327

[10] Marks DI Clifton-Hadley L Copland M Hussain J Menne TF McMillan A. In-vivo T-cell depleted reduced-intensity conditioned allogeneic haematopoietic stem-cell transplantation for patients with acute lymphoblastic leukaemia in first remission: results from the prospective, single-arm evaluation of the UKALL14 trial. Lancet Haematol (2022) 9(4):e276–88. doi: 10.1016/S2352-3026(22)00036-9 PMC8969058 35358442

[11] Fröbel J Landspersky T Percin G Schreck C Rahmig S Ori A. The hematopoietic bone marrow niche ecosystem. Front Cell Dev Biol (2021) 9:1958. doi: 10.3389/fcell.2021.705410 PMC8339972 34368155

[12] Bianco P. Bone and the hematopoietic niche: A tale of two stem cells. Blood. (2011) 117(20):5281–8. doi: 10.1182/blood-2011-01-315069 21406722

[13] Man Y Yao X Yang T Wang Y. Hematopoietic stem cell niche during homeostasis, malignancy, and bone marrow transplantation. Front Cell Dev Biol (2021) 9:14. doi: 10.3389/fcell.2021.621214 PMC7862549 33553181

[14] Sitnicka E Bryder D Theilgaard-Mönch K Buza-Vidas N Adolfsson J Jacobsen SEW. Key role of flt3 ligand in regulation of the common lymphoid progenitor but not in maintenance of the hematopoietic stem cell pool. Immunity. (2002) 17(4):463–72. doi: 10.1016/S1074-7613(02)00419-3 12387740

[15] Waskow C Paul S Haller C Gassmann M Rodewald HR. Viable c-KitW/W mutants reveal pivotal role for c-kit in the maintenance of lymphopoiesis. Immunity. (2002) 17(3):277–88. doi: 10.1016/S1074-7613(02)00386-2 12354381

[16] Tokoyoda K Egawa T Sugiyama T Choi BIL Nagasawa T. Cellular niches controlling b lymphocyte behavior within bone marrow during development. Immunity. (2004) 20(6):707–18. doi: 10.1016/j.immuni.2004.05.001 15189736

[17] Galán-Díez M Kousteni S. The osteoblastic niche in hematopoiesis and hematological myeloid malignancies. Curr Mol Biol Rep (2017) 3(2):53–62. doi: 10.1007/s40610-017-0055-9 29098141 PMC5662025

[18] Park S-Y Wolfram P Canty K Harley B Nombela-Arrieta C Pivarnik G. Focal adhesion kinase regulates the localization and retention of pro-b cells in bone marrow microenvironments. J Immunol (2013) 190(3):1094–102. doi: 10.4049/jimmunol.1202639 PMC3552136 23264658

[19] Borzillo GV Ashmun RA Sherr CJ. Macrophage lineage switching of murine early pre-b lymphoid cells expressing transduced fms genes. Mol Cell Biol (1990) 10(6):2703–14. doi: 10.1128/mcb.10.6.2703-2714.1990 PMC360630 2160584

[20] Gauthier L Rossi B Roux F Termine E Schiff C. Galectin-1 is a stromal cell ligand of the pre-b cell receptor (BCR) implicated in synapse formation between pre-b and stromal cells and in pre-BCR triggering. Proc Natl Acad Sci U S A. (2002) 99(20):13014–9. doi: 10.1073/pnas.202323999 PMC130578 12271131

[21] Medvinsky A Rybtsov S Taoudi S. Embryonic origin of the adult hematopoietic system: advances and questions. Development. (2011) 138(6):1017–31. doi: 10.1242/dev.040998 21343360

[22] Böiers C Carrelha J Lutteropp M Luc S Green JCA Azzoni E. Lymphomyeloid contribution of an immune-restricted progenitor emerging prior to definitive hematopoietic stem cells. Cell Stem Cell (2013) 13(5):535–48. doi: 10.1016/j.stem.2013.08.012 24054998

[23] Popescu DM Botting RA Stephenson E Green K Webb S Jardine L. Decoding human fetal liver haematopoiesis. Nat (2019) 574(7778):365–71. doi: 10.1038/s41586-019-1652-y PMC6861135 31597962

[24] O’Byrne S Elliott N Rice S Buck G Fordham N Garnett C. Discovery of a CD10-negative b-progenitor in human fetal life identifies unique ontogeny-related developmental programs. Blood. (2019) 134(13):1059–71. doi: 10.1182/blood.2019001289 31383639

[25] Dander E Palmi C D’amico G Cazzaniga G. The bone marrow niche in b-cell acute lymphoblastic leukemia: The role of microenvironment from pre-leukemia to overt leukemia. Int J Mol Sci (2021) 22(9):4426. doi: 10.3390/ijms22094426 33922612 PMC8122951

[26] Ford AM Palmi C Bueno C Hong D Cardus P Knight D. The TEL-AML1 leukemia fusion gene dysregulates the TGF-β pathway in early b lineage progenitor cells. J Clin Invest (2009) 119(4):826–36. doi: 10.1172/JCI36428 PMC2662549 19287094

[27] Torrano V Procter J Cardus P Greaves M Ford AM. ETV6-RUNX1 promotes survival of early b lineage progenitor cells *via* a dysregulated erythropoietin receptor. Blood. (2011) 118(18):4910–8. doi: 10.1182/blood-2011-05-354266 21900195

[28] Böiers C Richardson SE Laycock E Zriwil A Turati VA Brown J. A human IPS model implicates embryonic b-myeloid fate restriction as developmental susceptibility to b acute lymphoblastic leukemia-associated ETV6-RUNX1. Dev Cell (2018) 44(3):362–377.e7. doi: 10.1016/j.devcel.2017.12.005 29290585 PMC5807056

[29] Wray JP Deltcheva EM Boiers C Richardson S Chhetri JB Brown J. Regulome analysis in b-acute lymphoblastic leukemia exposes core binding factor addiction as a therapeutic vulnerability. Nat Commun (2022) 13(1):1–18. doi: 10.1038/s41467-022-34653-3 36411286 PMC9678885

[30] Gefen N Binder V Zaliova M Linka Y Morrow M Novosel A. Hsa-mir-125b-2 is highly expressed in childhood ETV6/RUNX1 (TEL/AML1) leukemias and confers survival advantage to growth inhibitory signals independent of p53. Leuk (2010) 24(1):89–96. doi: 10.1038/leu.2009.208 PMC2811577 19890372

[31] Christensen JL Wright DE Wagers AJ Weissman IL. Circulation and chemotaxis of fetal hematopoietic stem cells. PloS Biol (2004) 2(3):e75. doi: 10.1371/journal.pbio.0020075 15024423 PMC368169

[32] Potocnik AJ Brakebusch C Fässler R. Fetal and adult hematopoietic stem cells require β1 integrin function for colonizing fetal liver, spleen, and bone marrow. Immunity. (2000) 12(6):653–63. doi: 10.1016/S1074-7613(00)80216-2 10894165

[33] Bowie MB McKnight KD Kent DG McCaffrey L Hoodless PA Eaves CJ. Hematopoietic stem cells proliferate until after birth and show a reversible phase-specific engraftment defect. J Clin Invest (2006) 116(10):2808–16. doi: 10.1172/JCI28310 PMC1578623 17016561

[34] Lewis K Yoshimoto M Takebe T. Fetal liver hematopoiesis: from development to delivery. Stem Cell Res Ther (2021) 12(1):1–8. doi: 10.1186/s13287-021-02189-w 33597015 PMC7890853

[35] Palmi C Fazio G Savino AM Procter J Howell L Cazzaniga V. Cytoskeletal regulatory gene expression and migratory properties of b-cell progenitors are affected by the ETV6-RUNX1 rearrangement. Mol Cancer Res (2014) 12(12):1796–806. doi: 10.1158/1541-7786.MCR-14-0056-T 25061103

[36] Zhang H Cheng J Li Z Xi Y. Identification of hub genes and molecular mechanisms in infant acute lymphoblastic leukemia with MLL gene rearrangement. PeerJ. (2019) 2019(8):e7628. doi: 10.7717/peerj.7628 PMC6717502 31523525

[37] Mori H Colman SM Xiao Z Ford AM Healy LE Donaldson C. Chromosome translocations and covert leukemic clones are generated during normal fetal development. Proc Natl Acad Sci U S A. (2002) 99(12):8242–7. doi: 10.1073/pnas.112218799 PMC123052 12048236

[38] Schäfer D Olsen M Lähnemann D Stanulla M Slany R Schmiegelow K. Five percent of healthy newborns have an ETV6-RUNX1 fusion as revealed by DNA-based GIPFEL screening. Blood. (2018) 131(7):821–6. doi: 10.1182/blood-2017-09-808402 PMC5909885 29311095

[39] Zanetti C Kumar R Ender J Godavarthy PS Hartmann M Hey J. The age of the bone marrow microenvironment influences b-cell acute lymphoblastic leukemia progression *via* CXCR5-CXCL13. Blood. (2021) 138(19):1870–84. doi: 10.1182/blood.2021011557 PMC8767790 34424946

[40] Swaminathan S Klemm L Park E Papaemmanuil E Ford A Kweon SM. Mechanisms of clonal evolution in childhood acute lymphoblastic leukemia. Nat Immunol (2015) 16(7):766–74. doi: 10.1038/ni.3160 PMC4475638 25985233

[41] Kirkham CM Scott JNF Wang X Smith AL Kupinski AP Ford AM. Cut-and-Run: A distinct mechanism by which V(D)J recombination causes genome instability. Mol Cell (2019) 74(3):584–597.e9. doi: 10.1016/j.molcel.2019.02.025 30905508 PMC6509286

[42] Papaemmanuil E Rapado I Li Y Potter NE Wedge DC Tubio J. RAG-mediated recombination is the predominant driver of oncogenic rearrangement in ETV6-RUNX1 acute lymphoblastic leukemia. Nat Genet (2014) 46(2):116–25. doi: 10.1038/ng.2874 PMC3960636 24413735

[43] Chang JS Zhou M Buffler PA Chokkalingam AP Metayer C Wiemels JL. Profound deficit of IL10 at birth in children who develop childhood acute lymphoblastic leukemia. Cancer Epidemiol Biomarkers Prev (2011) 20(8):1736–40. doi: 10.1158/1055-9965.EPI-11-0162 PMC3257311 21653647

[44] Søegaard SH Rostgaard K Skogstrand K Wiemels JL Schmiegelow K Hjalgrim H. Neonatal inflammatory markers are associated with childhood b-cell precursor acute lymphoblastic leukemia. Cancer Res (2018) 78(18):5458–63. doi: 10.1158/0008-5472.CAN-18-0831 PMC7605595 30217873

[45] Cazzaniga G Bisanti L Randi G Deandrea S Bungaro S Pregliasco F. Possible role of pandemic AH1N1 swine flu virus in a childhood leukemia cluster. Leuk (2017) 31(8):1819–21. doi: 10.1038/leu.2017.127 PMC5542028 28446785

[46] Kroll ME Draper GJ Stiller CA Murphy MFG. Childhood leukemia incidence in Britain, 1974–2000: Time trends and possible relation to influenza epidemics. JNCI J Natl Cancer Inst (2006) 98(6):417–20. doi: 10.1093/jnci/djj095 16537835

[47] Li CK Zee B Lee J Chik KW Ha SY Lee V. Impact of SARS on development of childhood acute lymphoblastic leukaemia. Leukemia. (2007) 21(7):1353–6. doi: 10.1038/sj.leu.2404729 PMC7099337 17579654

[48] Greaves M. COVID-19 and childhood acute lymphoblastic leukemia. Pediatr Blood Cancer (2020) 67(12):e28481. doi: 10.1002/pbc.28481 32860652

[49] Rodríguez-Hernández G Opitz FV Delgado P Walter C Álvarez-Prado ÁF González-Herrero I. Infectious stimuli promote malignant b-cell acute lymphoblastic leukemia in the absence of AID. Nat Commun (2019) 10:5563. doi: 10.1038/s41467-019-13570-y 31804490 PMC6895129

[50] Martín-Lorenzo A Hauer J Vicente-Dueñas C Auer F González-Herrero I García-Ramírez I. Infection exposure is a causal factor in b-cell precursor acute lymphoblastic leukemia as a result of Pax5-inherited susceptibility. Cancer Discovery (2015) 5(12):1328–43. doi: 10.1158/2159-8290.CD-15-0892 26408659

[51] Isidro-Hernández M Mayado A Casado-García A Martínez-Cano J Palmi C Fazio G. Inhibition of inflammatory signaling in Pax5 mutant cells mitigates b-cell leukemogenesis. Sci Rep (2020) 10(1):1–14. doi: 10.1038/s41598-020-76206-y 33154497 PMC7644722

[52] Portale F Cricrì G Bresolin S Lupi M Gaspari S Silvestri D. ActivinA: a new leukemia-promoting factor conferring migratory advantage to b-cell precursor-acute lymphoblastic leukemic cells. Haematologica. (2019) 104(3):533–45. doi: 10.3324/haematol.2018.188664 PMC6395324 30262563

[53] Beneforti L Dander E Bresolin S Bueno C Acunzo D Bertagna M. Pro-inflammatory cytokines favor the emergence of ETV6-RUNX1-positive pre-leukemic cells in a model of mesenchymal niche. Br J Haematol (2020) 190(2):262–73. doi: 10.1111/bjh.16523 32118299

[54] Vicente-Duenãs C Janssen S Oldenburg M Auer F Lez-Herrero ISG Casado-García A. An intact gut microbiome protects genetically predisposed mice against leukemia. Blood. (2020) 136(18):2003–17. doi: 10.1182/blood.2019004381 PMC7694022 32911536

[55] Duan CW Shi J Chen J Wang B Yu YH Qin X. Leukemia propagating cells rebuild an evolving niche in response to therapy. Cancer Cell (2014) 25(6):778–93. doi: 10.1016/j.ccr.2014.04.015 24937459

[56] Balandrán JC Purizaca J Enciso J Dozal D Sandoval A Jiménez-Hernández E. Pro-inflammatory-related loss of CXCL12 niche promotes acute lymphoblastic leukemic progression at the expense of normal lymphopoiesis. Front Immunol (2017) 7:666. doi: 10.3389/fimmu.2016.00666 28111575 PMC5216624

[57] Rajakumar SA Danska JS. Bad to the bone: B cell acute lymphoblastic leukemia cells mediate bone destruction. Mol Cell Oncol (2020) 8(1):1835423. doi: 10.1080/2372355620201835423 33553597 PMC7849691

[58] Cheung LC Tickner J Hughes AM Skut P Howlett M Foley B. New therapeutic opportunities from dissecting the pre-b leukemia bone marrow microenvironment. Leukemia. (2018) 32:2326–38. doi: 10.1038/s41375-018-0144-7 PMC6224400 29740160

[59] Rajakumar SA Papp E Lee KK Grandal I Merico D Liu CC. B cell acute lymphoblastic leukemia cells mediate RANK-RANKL-dependent bone destruction. Sci Transl Med (2020) 12(561):5942. doi: 10.1126/scitranslmed.aba5942 32938796

[60] Fei F Joo EJ Tarighat SS Schiffer I Paz H Fabbri M. B-cell precursor acute lymphoblastic leukemia and stromal cells communicate through galectin-3. Oncotarget. (2015) 6(13):11378–94. doi: 10.18632/oncotarget.3409 PMC4484463 25869099

[61] Ma C Witkowski MT Harris J Dolgalev I Sreeram S Qian W. Leukemia-on-a-chip: Dissecting the chemoresistance mechanisms in b cell acute lymphoblastic leukemia bone marrow niche. Sci Adv (2020) 6(44):eaba5536. doi: 10.1126/sciadv.aba5536 33127669 PMC7608809

[62] Hsieh Y Gang EJ Geng H Park E Huantes S Chudziak D. Integrin alpha4 blockade sensitizes drug resistant pre-b acute lymphoblastic leukemia to chemotherapy. Blood. (2013) 121(10):1814–8. doi: 10.1182/blood-2012-01-406272 PMC3591800 23319569

[63] Burt R Dey A Aref S Aguiar M Akarca A Bailey K. Activated stromal cells transfer mitochondria to rescue acute lymphoblastic leukemia cells from oxidative stress. Blood. (2019) 134(17):1415–29. doi: 10.1182/blood.2019001398 PMC6856969 31501154

[64] Tucci J Chen T Margulis K Orgel E Paszkiewicz RL Cohen MD. Adipocytes provide fatty acids to acute lymphoblastic leukemia cells. Front Oncol (2021) 11:665763. doi: 10.3389/fonc.2021.665763 33968771 PMC8100891

[65] Sheng X Parmentier JH Tucci J Pei H Cortez-Toledo O Dieli-Conwright CM. Adipocytes sequester and metabolize the chemotherapeutic daunorubicin. Mol Cancer Res (2017) 15(12):1704–13. doi: 10.1158/1541-7786.MCR-17-0338 PMC5726435 29117945

[66] Lee M Hamilton JAG Talekar GR Ross AJ Michael L Rupji M. Obesity-induced galectin-9 is a therapeutic target in b-cell acute lymphoblastic leukemia. Nat Commun (2022) 13(1):1157. doi: 10.1038/s41467-022-28839-y 35241678 PMC8894417

[67] Heydt Q Xintaropoulou C Clear A Austin M Pislariu I Miraki-Moud F. Adipocytes disrupt the translational programme of acute lymphoblastic leukaemia to favour tumour survival and persistence. Nat Commun (2021) 12(1):1–18. doi: 10.1038/s41467-021-25540-4 34535653 PMC8448863

[68] Duell J Dittrich M Bedke T Mueller T Eisele F Rosenwald A. Frequency of regulatory T cells determines the outcome of the T-cell-engaging antibody blinatumomab in patients with b-precursor ALL. Leuk (2017) 31(10):2181–90. doi: 10.1038/leu.2017.41 PMC5629361 28119525

[69] Chao MP Alizadeh AA Tang C Jan M Weissman-Tsukamoto R Zhao F. Therapeutic antibody targeting of CD47 eliminates human acute lymphoblastic leukemia. Cancer Res (2011) 71(4):1374–84. doi: 10.1158/0008-5472.CAN-10-2238 PMC3041855 21177380

[70] Dave DS Leppert JT Rajfer J. Is the testis a chemo-privileged site? is there a blood-testis barrier? Rev Urol (2007) 9(1):28. 17396169 PMC1831524

[71] Akers SM Rellick SL Fortney JE Gibson LF. Cellular elements of the subarachnoid space promote ALL survival during chemotherapy. Leuk Res (2011) 35(6):705–11. doi: 10.1016/j.leukres.2010.12.031 PMC3099259 21269691

[72] Frishman-Levy L Shemesh A Bar-Sinai A Ma C Ni Z Frenkel S. Central nervous system acute lymphoblastic leukemia: role of natural killer cells. Blood. (2015) 125(22):3420–31. doi: 10.1182/blood-2014-08-595108 PMC7265786 25896649

[73] Juarez J Dela Pena A Baraz R Hewson J Khoo M Cisterne A. CXCR4 antagonists mobilize childhood acute lymphoblastic leukemia cells into the peripheral blood and inhibit engraftment. Leuk (2007) 21(6):1249–57. doi: 10.1038/sj.leu.2404684 17410186

[74] Kato I Niwa A Heike T Fujino H Saito MK Umeda K. Identification of hepatic niche harboring human acute lymphoblastic leukemic cells *via* the SDF-1/CXCR4 axis. PloS One (2011) 6(11):e27042. doi: 10.1371/journal.pone.0027042 22069486 PMC3206061

[75] Kato I Nishinaka Y Nakamura M Akarca AU Niwa A Ozawa H. Hypoxic adaptation of leukemic cells infiltrating the CNS affords a therapeutic strategy targeting VEGFA. Blood. (2017) 129(23):3126–9. doi: 10.1182/blood-2016-06-721712 PMC5473134 28424164

[76] Kallmann BA Wagner S Hummel V Buttmann M Bayas A Tonn JC. Characteristic gene expression profile of primary human cerebral endothelial cells. FASEB J (2002) 16(6):589–91. doi: 10.1096/fj.01-0594fje 11919163

[77] Williams MTS Yousafzai YM Elder A Rehe K Bomken S Frishman-Levy L. The ability to cross the blood-cerebrospinal fluid barrier is a generic property of acute lymphoblastic leukemia blasts. Blood. (2016) 127(16):1998–2006. doi: 10.1182/blood-2015-08-665034 26869395

[78] Lenk L Carlet M Vogiatzi F Spory L Winterberg D Cousins A. CD79a promotes CNS-infiltration and leukemia engraftment in pediatric b-cell precursor acute lymphoblastic leukemia. Commun Biol (2021) 4(1):73. doi: 10.1038/s42003-020-01591-z 33452446 PMC7810877

[79] Alsadeq A Lenk L Vadakumchery A Cousins A Vokuhl C Khadour A. IL7R is associated with CNS infiltration and relapse in pediatric b-cell precursor acute lymphoblastic leukemia. Blood. (2018) 132(15):1614. doi: 10.1182/blood-2018-04-844209 30154115 PMC6238156

[80] Kinjyo I Bragin D Grattan R Winter SS Wilson BS. Leukemia-derived exosomes and cytokines pave the way for entry into the brain. J Leukoc Biol (2019) 105(4):741–53. doi: 10.1002/JLB.3A0218-054R PMC7200047 30702754

[81] Cousins A Olivares O Markert E Manoharan A Bubnova X Bresolin S. Central nervous system involvement in childhood acute lymphoblastic leukemia is linked to upregulation of cholesterol biosynthetic pathways. Leukemia. (2022) 36(12):2903–7. doi: 10.1038/s41375-022-01722-x PMC9712090 36289348

[82] Savino AM Fernandes SI Olivares O Zemlyansky A Cousins A Markert EK. Metabolic adaptation of acute lymphoblastic leukemia to the central nervous system microenvironment is dependent on stearoyl CoA desaturase. Nat cancer (2020) 1(10):998–1009. doi: 10.1038/s43018-020-00115-2 33479702 PMC7116605

[83] Turati VA Guerra-Assunção JA Potter NE Gupta R Ecker S Daneviciute A. Chemotherapy induces canalization of cell state in childhood b-cell precursor acute lymphoblastic leukemia. Nat Cancer (2021) 2(8):835–52. doi: 10.1038/s43018-021-00219-3 PMC7611923 34734190

[84] Loukas I Simeoni F Milan M Inglese P Patel H Goldstone R. Selective advantage of epigenetically disrupted cancer cells via phenotypic inertia. Cancer Cell (2023) 41(1):70–87.e14. doi: 10.1016/j.ccell.2022.10.002 36332625

[85] Holmfeldt L Wei L Diaz-Flores E Walsh M Zhang J Ding L. The genomic landscape of hypodiploid acute lymphoblastic leukemia. Nat Genet (2013) 45:242–52. doi: 10.1038/ng.2532 PMC3919793 23334668

[86] Inthal A Zeitlhofer P Zeginigg M Morak M Grausenburger R Fronkova E. CREBBP HAT domain mutations prevail in relapse cases of high hyperdiploid childhood acute lymphoblastic leukemia. Leuk (2012) 26(8):1797–803. doi: 10.1038/leu.2012.60 PMC4194312 22388726

[87] Gang EJ Hsieh YT Pham J Zhao Y Nguyen C Huantes S. Small-molecule inhibition of CBP/catenin interactions eliminates drug-resistant clones in acute lymphoblastic leukemia. Oncogene. (2014) 33(17):2169–78. doi: 10.1038/onc.2013.169 PMC3994178 23728349

[88] Dixon ZA Nicholson L Zeppetzauer M Matheson E Sinclair P Harrison CJ. CREBBP knockdown enhances RAS/RAF/MEK/ERK signaling in ras pathway mutated acute lymphoblastic leukemia but does not modulate chemotherapeutic response. Haematologica. (2017) 102(4):736–45. doi: 10.3324/haematol.2016.145177 PMC5395114 27979926

[89] Pal D Blair H Parker J Hockney S Beckett M Singh M. hiPSC-derived bone marrow milieu identifies a clinically actionable driver of niche-mediated treatment resistance in leukemia. Cell Rep Med (2022) 3(8):100717. doi: 10.1016/j.xcrm.2022.100717 35977468 PMC9418860

[90] Shen ZH Zeng DF Wang XY Ma YY Zhang X Kong PY. Targeting of the leukemia microenvironment by c(RGDfV) overcomes the resistance to chemotherapy in acute myeloid leukemia in biomimetic polystyrene scaffolds. Oncol Lett (2016) 12(5):3278–84. doi: 10.3892/ol.2016.5042 PMC5103942 27899994

[91] Greaves M. A causal mechanism for childhood acute lymphoblastic leukaemia. Nat Rev Cancer (2018) 18(8):471–84. doi: 10.1038/s41568-018-0015-6 PMC6986894 29784935

